# Trends in urological emergencies in the Era of COVID-19

**DOI:** 10.1590/S1677-5538.IBJU.2020.1092

**Published:** 2021-04-20

**Authors:** Michael Frumer, Shachar M. Aharony, Ohad Shoshany, Daniel Kedar, Jack Baniel, Shay Golan

**Affiliations:** 1 Rabin Medical Center Department of Urology Petach Tikva Israel Department of Urology, Rabin Medical Center, Petach Tikva, Israel; 2 Tel Aviv University Sackler Faculty of Medicine Tel Aviv Israel Sackler Faculty of Medicine, Tel Aviv University, Tel Aviv, Israel

**Keywords:** trends [Subheading], Emergencies, Covid-19

## Abstract

**Purpose:**

To evaluate trends in emergency room (ER) urological conditions during COVID-19 pandemic lockdown.

**Materials and Methods:**

Retrospective analyses of renal colic, hematuria, and urinary retention in ER's admissions of a tertiary hospital during the lockdown period (March 19 to May 4, 2020) in Israel. Patient's demographics and clinical characteristics were compared to those in corresponding periods during 2017-2019, with estimated changes in ER arrival and waiting times, utilization of imaging tests, numbers of hospitalizations, and urgent procedure rates.

**Results:**

The number of ER visits for renal colic, hematuria, and urinary retention decreased by 37%, from an average of 451 (2017-2019) to 261 patients (2020). Clinical severity was similar between groups, with no major differences in patient's age, vital signs, or laboratory results. The proportion of ER visits during night hours increased significantly during lockdown (44.8% vs. 34.2%, p=0.002). There was a decrease in renal colic admission rate from 19.8% to 8.4% (p=0.001) without differences in urgent procedures rates, while the 30-day revisit rate decreased from 15.8% to 10.3% during lockdown (p=0.02).

**Conclusions:**

General lockdown was accompanied by a significant decrease in common urological presentations to the ER. This change occurred across the clinical severity spectrum of renal colic, hematuria, and urinary retention. In the short term, it appears that patients who sought treatment did not suffer from complications that could be attributed to late arrival or delay in treatment. The long-term implications of abstinence from seeking emergent care are not known and require further investigation.

## INTRODUCTION

Since the onset in December 2019, the coronavirus disease 2019 (COVID-19) outbreak has spread globally (1). Many countries have declared a state of emergency and imposed lockdown restrictions to reduce transmission of the virus. On March 11, 2020, Israel began enforcing social distancing, and a full national lockdown was imposed from March 19 to May 4. During this period, community medical services were limited with a reduction in the number of outpatient clinic sessions, and telemedicine utilization increased significantly (2). Public health messaging advised to avoid non-urgent health care to accommodate surges in COVID-19 cases. While a decrease in visits to the emergency room (ER) during lockdown was reported (3), its consequences are unclear. Avoiding medical care can be harmful to health and even life-threatening, especially in medical situations in which the symptoms are vague, not involving sharp pain or an obvious threatening condition. We hypothesized that the number of urological visits to the ER during the lockdown decreased and their clinical severity increased, compared to corresponding periods in previous years. While the benefits of full lockdown in reducing the spread of the virus have already been seen in several countries, the short-term implications of lockdown restrictions on other medical problems, including urological emergencies, require further research.

## MATERIALS AND METHODS

After approval by the Institutional Review Board (0419-20-RMC), we retrospectively reviewed our institutional medical records to identify all patients who attended the ER for renal colic, hematuria, and urinary retention during the lockdown period. Any patient, COVID-19 suspected and not suspected, could access the emergency room at our institution during the lockdown period. Patient's demographics and clinical characteristics were compared between the lockdown period with average results during corresponding periods in 2017-2019.

To estimate the severity of each acute illness, vital signs and laboratory and imaging results were recorded. These included hemoglobin (Hb), hematocrit, white blood cells (WBC), electrolytes, creatinine, C-reactive protein (CRP), urinalysis, and residual urine volume. Computed tomography scan (CT)/ultrasound (US) performance rates were recorded during renal colic visit evaluations. In addition, we documented ER waiting times and rates of hospitalizations and surgeries performed within a week. In order to estimate ER activity throughout the day, we distinguished between daytime (8 AM-8 PM) and nighttime arrivals.

Basic descriptive statistics for categorical and continuous variables were determined, with continuous variables reported as median and interquartile ranges unless otherwise stated. Comparative tests (Pearson's chi-squared test for categorical variables, the Mann-Whitney test for ordinal and continuous variables) were used to compare between the periods. All statistical analyses were performed using SPSS software, version 25.0 (SPSS Inc., Chicago, IL, USA), with a two-sided significance level set at p <0.05. All methods were performed in accordance with relevant guidelines and regulations.

## RESULTS

Of 15.217 visits to the ER during the lockdown period, 167 (1.1%), 55 (0.36%), and 39 (0.26%) patients presented with renal colic, gross hematuria, and urinary retention, respectively. The number of these urological complaints decreased by 37%, from an average of 451 (2017-2019) to 261 patients (2020), however, their proportion out of total ER visits was not statistically different (1.9% vs. 1.7%, p=0.15).

Urological visits during night hours increased from 34.2% (427/1246) in 2017-2019 to 44.8% (117/261) in 2020, p=0.002. While median time to triage was shorter than in 2020 (11 (IQR: 6-20) vs. 13 (IQR: 8-23) minutes, respectively, p <0.001), the total length of stay in the ER was longer (5.3 (IQR: 3.7-7.2) vs. 4.8 (IQR: 3.2-6.8) hours, respectively, p=0.003).

There were no significant differences in admission rates (14.9% in 2020 vs. 19.4% in previous years, p=0.09), or in the rate of surgeries performed within a week (6.1% in 2020 vs. 6.3% in previous years, p=0.88). However, we noticed a decrease in the 30-day ER revisit rate, which dropped from 15.8% in previous years to 10.3% in 2020 (p=0.02).

### 

#### Renal colic

While renal colic remained the most prevalent of the three urological emergencies, we noticed a 21% decrease from an average of 212 visits in 2017-9, to 167 visits during the lockdown period. The proportion of renal colic among all ER visitors did not change (1.1% in 2017-9 vs. 0.96% in 2020, p=0.9). CRP levels were higher among visitors during the lockdown (0.47mg/L (IQR: 0.2-1.5) vs. 0.4mg/L (IQR: 0.16-1), p=0.03), but other laboratory results such as creatinine, WBC, and leukocyturia, did not differ statistically. There were no differences in patient's age or vital signs between the study periods. CT and ultrasound were performed less frequently during lockdown (41% vs. 51%, P=0.017), but the relative utilization of CT was higher (97% vs. 86.5%, P=0.014). The rate of admission was lower during lockdown (8.4% vs. 19.8%, p=0.001), and CRP levels were higher among hospitalized patients (4.9mg/L vs. 0.8mg/L, p=0.005) (Table-1).

**Table 1:** Renal colic.

Parameter		2020	2017-9	*P* value
Total visits, 19 March to 4 May, per year		167	212	
Age, years (IQR)		47 (36-58)	47 (36-58)	0.9
**Sex**				
	Male (%)	123 (73)	160 (76)	0.5
	Female (%)	44 (27)	52 (24)	
Time to first nurse, minutes (IQR)		9 (5-15)	12 (8-19)	<0.001
Lengths of stay in the ER, hours (IQR)		5.5 (4-7.3)	5.1 (3.6-7.1)	0.12
Systolic blood pressure, mmHg (IQR)		136 (120-157)	134 (123-149)	0.5
Diastolic blood pressure, mmHg (IQR)		81.5 (74-95)	81 (72-90)	0.8
Pulse, beats per minute (IQR)		80 (71-90)	78 (70-88)	0.18
Fever, ^0^C (IQR)		36.7 (36.5-36.9)	36.7 (36.5-36.9)	0.9
Urea, serum, mg/dL (IQR)		32 (27-41)	34 (28-41)	0.2
Creatinine, serum, mg/dL (IQR)		1.04 (0.84-1.25)	1.02 (0.85-1.2)	0.5
WBC, serum, x 10^3^ /μL (IQR)		10 (7.6-12.8)	9.7 (7.7-12)	0.15
CRP, serum, mg/L (IQR)		0.47 (0.2-1.5)	0.4 (0.16-1)	0.03
Leukocyturia		10.2%	8.2%	0.4
Nitrituria		4.3%	3%	0.4
Imaging performance rate		40.7%	51.1%	0.017
**Type of imaging study**				0.014
	US (%)	2 (3)	46 (13.5)
	CT (%)	67 (97)	296 (86.5)
Rate of admission (%)		14 (8.4)	42 (19.8)	0.001
Length of stay, days (IQR)		1.5 (1-3.5)	2 (1-3)	0.6
Night time (%)		73 (43.2)	82 (38.6)	0.25
30-day ER's revisit rate (%)		17 (10.1)	29 (13.6)	0.24
Surgeries within a week (%)		13 (8)	21 (10)	0.4

**IQR** = interquartile range; **ER** = emergency room; **WBC** = white blood cells; **CRP** = C-reactive protein; **US** = ultrasound; **CT** = computed tomography

#### Hematuria

The number of patients presented with hematuria was 55 in 2020, compared with an average of 84 in 2017-9. The proportion of patients who arrived during night hours was higher in 2020 (41.8% vs. 27%, p=0.026). Looking into severity parameters, no significant differences were found in vital signs or laboratory findings. The 2020 admission rate was 29% (16/55), similar to 23% (19/84.3) in the previous years (p=0.3).

Although we did not find differences in hospitalized patient's characteristics, the length of stay was significantly shorter: 2.5 days (IQR: 2-5.2) in 2020 vs. 4 days (IQR: 3-9) in previous years (p=0.03) (Table-2).

**Table 2:** Hematuria

Parameter		2020	2017-9	*P* value
Total visits, 19 March to 4 May (2020 or average during 2017-2019)		55	84.3	
Age, years (IQR)		77 (65-83)	72 (63-83)	0.08
**Sex**				
	Male (%)	41 (74)	68 (81)	0.2
	Female (%)	14 (26)	16.3 (19)	
*Time to first nurse*, minutes *(IQR)*		13 (7-23)	15 (9-25)	0.24
Length of stay in the ER, hours (IQR)		5.5 (3.7-7.3)	4.5 (3.2-6.3)	0.07
Systolic blood pressure, mmHg (IQR)		142 (126-159)	138 (125-152)	0.51
Diastolic blood pressure, mmHg (IQR)		74 (66-89)	74 (67-85)	0.85
Pulse, beats per minute (IQR)		82 (69-94)	79 (68-90)	0.57
Fever, ^0^C (IQR)		36.9 (36.4-37.3)	36.7 (36.5-36.9)	0.39
Hemoglobin, serum, mg/dL (IQR)		12.9 (11-14.1)	12.6 (10.6-14)	0.47
Hematocrit, serum, mg/dL (IQR)		38.8 (33-42)	39 (34-42.5)	0.83
CRP, serum, mg/L (IQR)		0.84 (0.35-3.2)	0.65 (0.23-1.5)	0.07
Rate of admission (%)		16 (29)	19 (23)	0.3
Length of stay, days (IQR)		2.5 (2-5.2)	4 (3-9)	0.03
Nighttime (%)		23 (41.8)	23 (27)	0.026
30-day ER's revisit rate (%)		5 (9)	13 (15)	0.26
Surgeries within a week (%)		3 (5.5)	3.3 (4)	0.6

**IQR** = interquartile range; **ER** = emergency room; **CRP** = C-reactive protein

#### Urinary retention

The number of visits to the ER for urinary retention decreased by 67% from an average of 119 to 39 visits, and their proportion among all ER visitors dropped significantly from 0.54% (119/22.071) in previous years to 0.26% (39/15.217) during the lockdown (p <0.001). ER visitors during lockdown were older (83 years (IQR: 70-87) vs. 71 years (IQR: 64-83), p=0.001) but presented with similar vital signs and lab results. The proportion of visits during night hours was significantly higher (53.8% vs. 31.9%, p=0.006). No significant differences were found in admission rates or admitted patient's characteristics (Table-3).

**Table 3:** Urinary retention.

Parameter		2020	2017-9	*P* value
Total visits, 19 March to 4 May (2020 or average during 2017-2019)		39	119	
Age, years (IQR)		83 (70-87)	71 (64-83)	0.001
**Sex**				
	Male (%)	32 (82)	97.5 (82)	0.99
	Female (%)	7 (18)	21.5 (18)	
Time to first nurse, minutes (IQR)		19 (9-30)	16 (9-27)	0.4
Lengths of stay in the ER, hours (IQR)		4.1 (2.8-6.1)	4.4 (2.7-6.3)	0.8
Systolic blood pressure, mmHg (IQR)		140 (123-158)	138 (124-150)	0.6
Diastolic blood pressure, mmHg (IQR)		71 (65-85)	77 (68-88)	0.2
Pulse, beats per minute (IQR)		75 (65-85)	84 (74-97)	0.001
Fever, °C (IQR)		36.6 (36.4-36.9)	36.7 (36.5-36.9)	0.5
Residual urine volume, mL (IQR)		600 (200-800)	600 (280-900)	0.8
Urea, serum, mg/dL (IQR)		50 (31-68)	40 (30-56)	0.1
Creatinine, serum, mg/dL (IQR)		1.1 (0.9-1.8)	1 (0.8-1.2)	0.06
WBC, serum, x 10^3^ /μL (IQR)		8 (6.3-9.8)	8.8 (7-11.5)	0.08
CRP, serum, mg/L (IQR)		1.1 (0.6-3.2)	0.8 (0.3-3-5)	0.4
*Potassium disorders		3%	10%	0.33
**Sodium disorders		32%	21%	0.15
Rate of admission (%)		9 (23)	20 (17)	0.3
Length of stay, days (IQR)		3 (1-8)	4 (1-7.2)	0.7
Nighttime (%)		21 (53.8)	38 (31.9)	0.006
30-day ER's revisit rate (%)		5 (12.8)	24 (20)	0.26
Surgeries within a week (%)		0 (0)	1.7 (1.4)	1

* 3.6 > Potassium or Potassium > 5.2 mmol/L

** 135 > Sodium or Sodium > 145 mmol/L

**IQR** = interquartile range; **ER** = emergency room; **WBC** = white blood cells; **CRP** = C-reactive protein

## DISCUSSION

We evaluated ER visits for urological emergencies during COVID-19 lockdown and found several patterns that characterized this period. We noticed a sharp decrease in renal colic, hematuria, and urinary retention visits and revisits compared to previous years, but the proportion of these urological emergencies among all ER visits and their level of severity did not change. Our findings suggest that patients with urological emergencies across the severity spectrum abstained from prompt medical workup and treatment.

A few studies have investigated trends in urological emergencies during COVID-19 lockdown. Studies from Italy have shown up to a 60% decrease in urological admissions to the ER (4–6). There are several possible explanations for the milder (37%) reduction in urological visits in our study. The virus incidence rate was relatively low in Israel during the lockdown period when there was a 24-fold increase in the number of COVID-19 cases (677 to 16.246 cases) over seven weeks. In Italy, a 108-fold increase from 229 to 24.762 cases was recorded during just three weeks of restrictions (4, 7). The rapid increase in severe COVID-19 cases led to a serious healthcare crisis in Italy and extreme decrease in ER urological emergencies. In Israel, non-governmental health care organizations provided health services for COVID-19 patients at home and in designated hotels. This widespread primary care response and moderate virus incidence rate enabled healthcare facilities to maintain resources for non-COVID-19 emergent care (8).

Despite a reduction in patient load, ER time to triage was similar to previous years. This might be related to a parallel decrease in the number of ER healthcare providers (9). Recent reports have shown that many ER physicians and nurses were forced into isolation or transferred to emerging COVID-19 wards (10–12). Moreover, the proportion of patients arriving during night hours was higher compared with previous years. This is in line with a report by Hughes et al. who showed that night-to-day hours ER visits increased during COVID-19 restrictions from 35% to 45% (13). While this may have reflected the patient's desires to avoid daytime mobilization during the lockdown, the medical team is reduced during night hours and this could explain why ER times were not significantly shorter.

Another finding is the change in imaging utilization for patients with renal colic. Overall, US and CT were performed less frequently during lockdown compared to previous years. This might be explained by efforts to limit patient's mobilization across the hospital. Interestingly, the relative use of US also decreased, possibly as it requires direct contact with the physician or technician, which was avoided as much as possible during the pandemic (14). In addition, CT is more accurate than US for demonstrating nephrolithiasis, and more commonly provides a decisive result. This was shown by Smith-Bindman et al. who found that 27% of renal colic patients, initially evaluated with US by radiologists, also required CT during their primary workup (15).

Looking into severity parameters, we found no consistent clinical or laboratory findings that support a change in urological ER patient's severity profile during the lockdown. Although CRP levels were statistically higher in renal colic patients, the clinical significance of this change (from 0.4 to 0.47mg/L) is questionable. Our findings support several recent reports. In their multicenter study, Rajwa et al. found no differences in the laboratory parameters of 3883 patients with renal colic, hematuria, or urinary retention between the 2020 pandemic and 2019 reference periods in Poland (16). In a comparison of patient's characteristics before any restrictions and during the severe lockdown, no differences were found in levels of creatinine, Hb, CRP, and WBC. In a cohort of 80 patients presenting to the ER with renal colic during the lockdown in Italy, Flammia et al. found higher serum creatinine levels compared with the parallel period in 2019 (2.9 vs. 1.2mg/dL, p=0.026). However, WBC level, rate of urinary tract infection, hydronephrosis, and rate of urgent kidney drainage were similar (17). A single study from Turkey showed a more severe clinical profile of 149 renal colic patients during the COVID-19 restrictions period (18). The authors reported increased serum creatinine levels (1.9 vs. 1.15mg/dL), WBC counts (12.45 vs. 8.21 x 103/μL), and rates of ESBL (+) bacterial infection (37% vs. 13%) (p=0.034, 0.005, and <0.001, respectively). The authors identified mobilization restrictions, public anxiety, and telehealth availability as potential contributors to their results.

The hospital admission-to-ER visits ratio in our study was similar to previous years except for patients with renal colic. While we found a ratio of 8.4%, the reported admission ratios for acute renal colic in the pre-COVID 19 era were 8%-20% (15, 19-22). We did not find differences in admitted patient's vital signs and lab results between the periods. It is reasonable to assume that admissions of stable patients for pain control occurred less frequently during the lockdown. Patient's desire to avoid exposure to the virus, and staff obligation to ensure the capacity to accommodate surges in COVID-19, contributed to a more liberal discharge policy (23). This is in line with a recent study on patient's perspectives during the pandemic, which reported that even uro-oncology patients prefer to postpone surgeries. The risk of contracting the virus during hospitalization was perceived by them as more dangerous than the postponement (24).

Our findings that urological patients across the clinical severity spectrum avoided ER visits raise several concerns. A previous pre-COVID-19 area study reported that approximately 10% of patients with hematuria have an associated life-threatening disease (25). It was also reported that a delay in bladder cancer diagnosis is associated with an increased risk of death from the disease (26). While renal colic is a common problem, a combination of obstructive stone and infection is a potentially life-threatening situation. It was recently shown that a delay of two or more days in renal decompression increased mortality by 30% in patients with obstructive pyelonephritis (27). Because we did not find any selection in patients who arrived at the ER, at least a portion of those who refrained from medical evaluation at the ER may be subjected to those and other risks. Although COVID-19 pandemic had detrimental effects on the delivery of health care, it also offers opportunities to improve access. Several studies were published over the last year, pointing to the advantages of telemedicine, virtual care, and tele-monitoring in increasing access to expertise without increasing costs (28, 29). More data on the long-term efficacy and safety of telehealth are necessary, but as for the short term, it appears to solve problems of limitations in mobility and to reduce unnecessary visits and the risk of viral transmission (30).

There are several limitations to this study. This is a single-center, retrospective study. Local factors may have affected the results. Data regarding symptoms onset or any continuous treatment at a different medical institution were not available. Despite these limitations, detailed data about the clinical severity and management of the common urological emergencies was provided. In terms of public health management, information at different arrival times regarding availability of ER staff, various imaging modalities, and operating rooms in a tertiary hospital during COVID-19 lockdown were presented.

While COVID-19 remains a serious medical problem and its implications on other medical situations are not clear, some implications on urological emergencies can be learned from this study.

## CONCLUSION

General lockdown due to COVID-19 was accompanied by a significant decrease in common urological presentations to the ER. These changes occurred across the clinical severity spectrum of renal colic, hematuria, and urinary retention. In the short term, it appears that patients who sought treatment did not suffer from complications that could be attributed to late arrival or delay in treatment. Further studies are required to evaluate the long-term implications of abstinence from seeking emergent care for these urological presentations.
